# Supplementary material for: Genome-wide discovery and phenotyping of non-coding transcripts in A. fumigatus reveals lncRNAs with a role in antifungal drug sensitivity
Source: Nat Commun. 2026 Feb 11;17:1832. doi: 10.1038/s41467-026-68543-9 (PMC12921299; doi:10.1038/s41467-026-68543-9)
Supplement: Supplementary file 1 — Supplementary Information [file 41467_2026_68543_MOESM1_ESM.pdf]

Supplementary Figures

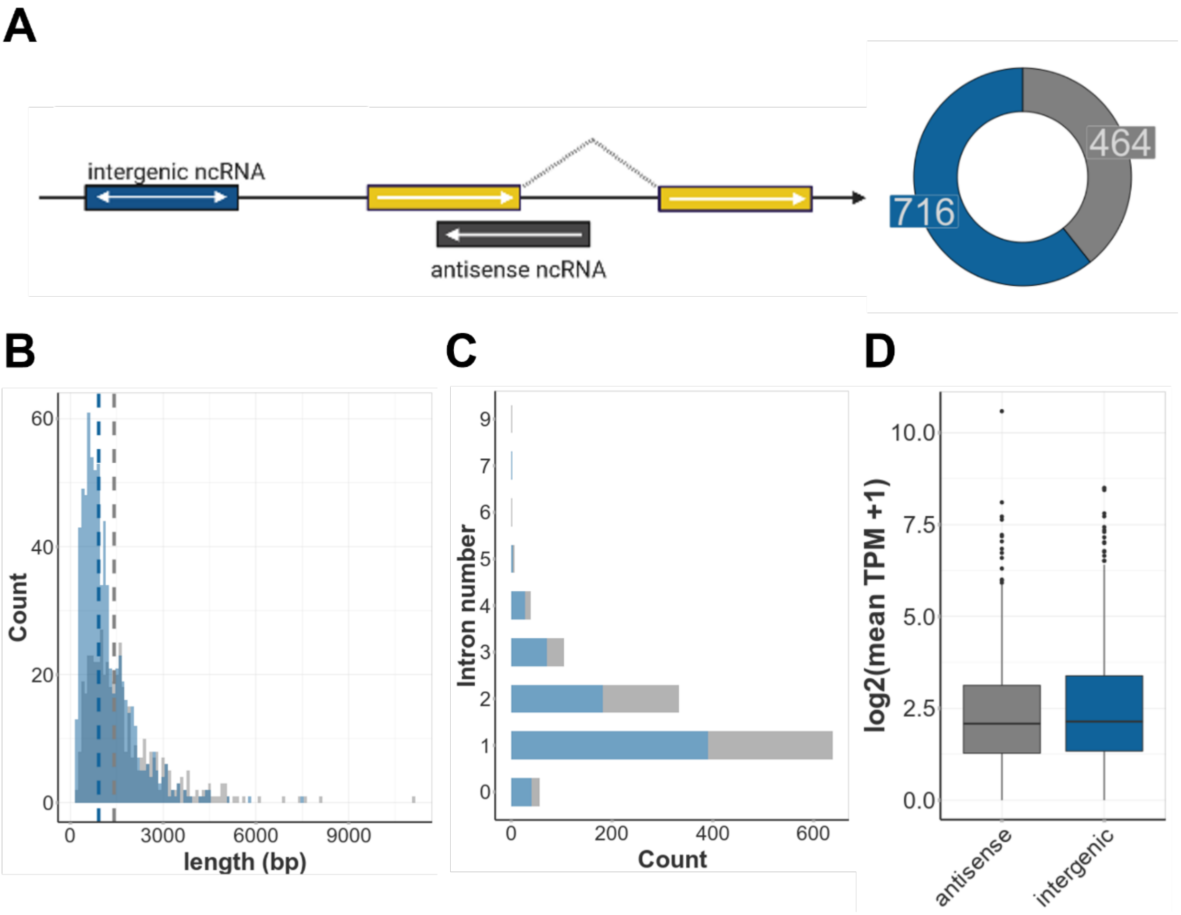

Supplementary Figure 1. *A. fumigatus* A1163 Noncoding RNA candidates predicted by automated pipeline. **A.** Genomic position of lncRNA with respect to coding genes (lncRNA class). Novel lncRNAs are predominantly intergenic (shown in blue, 60.7%), with only 39.3% being antisense (grey). **B.** Distribution of lncRNA length ranges between 207 bp and ~11.1 kb. Median lengths for intergenic and antisense lncRNAs (indicated by dotted lines) are 0.9 kb and 1.4 kb, respectively. **C.** Intron number ranges between 0-9, with a median of 1 intron per lncRNA. **D.** Expression levels displayed as log2 (mean TPM+1) of lncRNAs in untreated samples ranges between 0 and ~1500 TPM. The majority (n=724, 61.4%) of lncRNAs have TPM values below 5 (median TPM of 3.4 and 3.2 for intergenic and antisense, respectively). Boxplot centre lines indicate the median. The upper and lower hinges represent the upper quartile (Q3) and lower quartile (Q1), respectively. The upper and lower whiskers represent 1.5 times the interquartile range (IQR) above Q3 and below Q1, respectively.

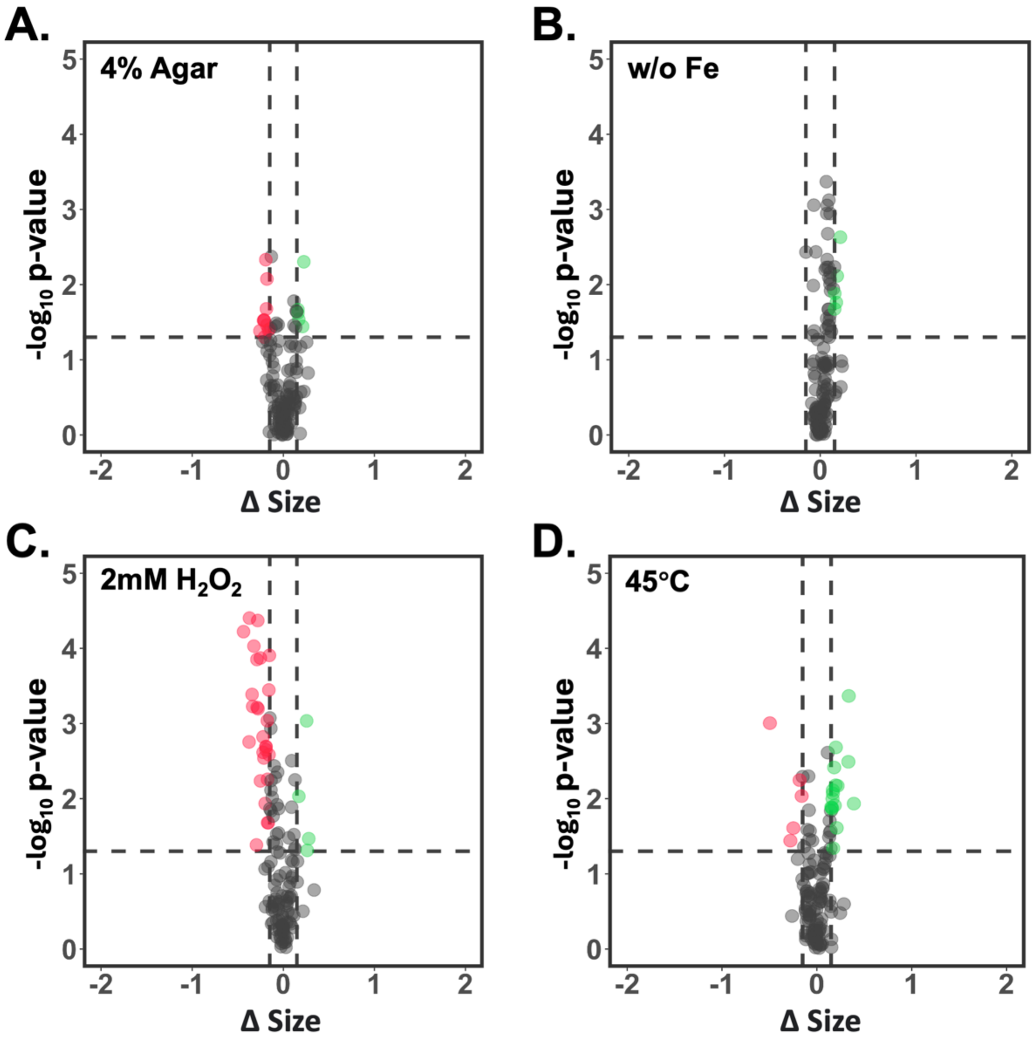

a

Supplementary Figure 2. Volcano plot exhibiting condition-dependent fitness changes of lncRNA mutants. The changes in fitness, reflected by relative growth rate of the lncRNA knockouts, was investigated in four conditions, including firm media (4% agar, panel A), iron-starved conditions (panel B), oxide stress (2mM H_2O_2, Panel C) and elevated temperature (45°C, panel D) in a high throughput screen. The growth of mutant in each condition was compared with their respective growth in standard condition AMM. The x-axis represents the normalised size change (Δ size) compared with the standard condition, and the y-axis represents the -$\log_{10}$ p-value. LncRNAs with a significant change in expression ($p \leq 0.05$; $|\Delta \text{ size}| >= 0.15$) are shown as red dots (fitness impaired) or green dots (fitness gain). Source data are provided with this paper.

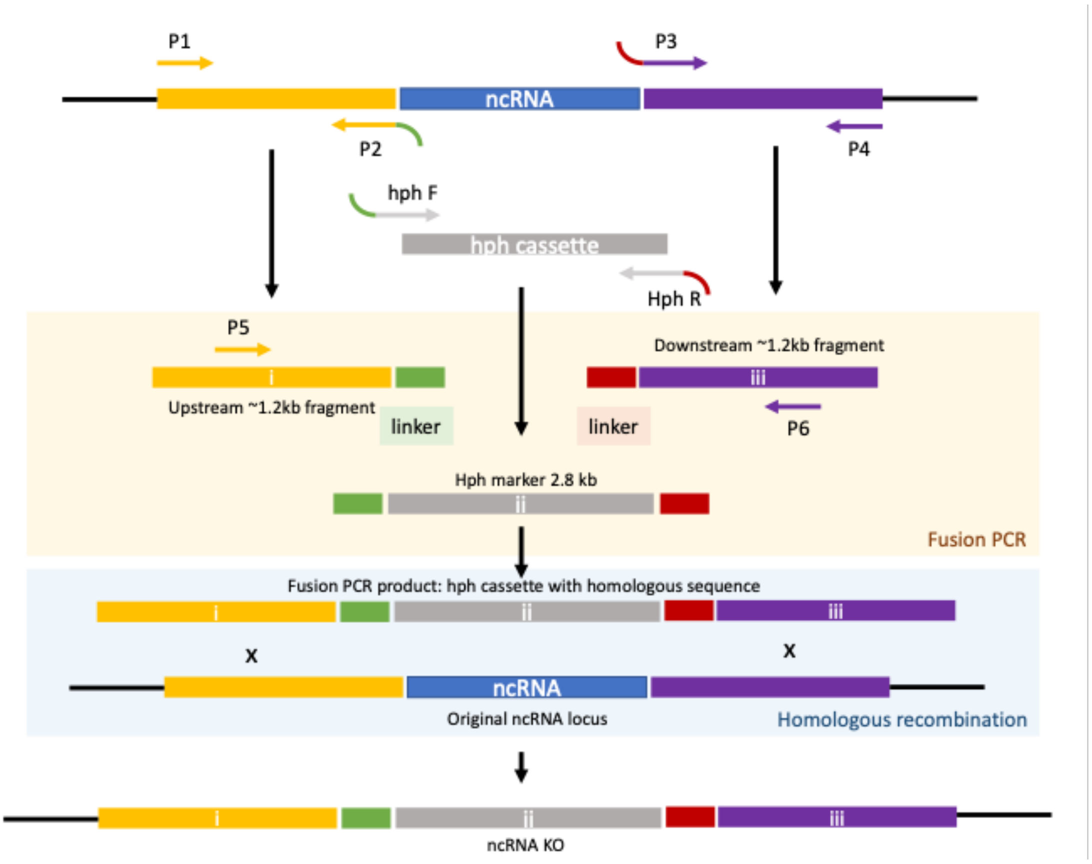

Supplementary Figure 3. Strategies used for the lncRNA deletions via fusion PCR and homologous recombination as previously described[39]. Upstream and downstream fragments are amplified using the primers pairs P1 and P2 (upstream) and P3 and P4 (downstream). The selective marker cassette (hph) is amplified using hph F and hph R primers and pAN7-1 plasmid was used as template. Linkers sequences are incorporated into the P2 and P3 primers to promote fusion with the marker cassette. Using nested primers P5 and P6, the upstream and downstream fragments are fused to the hph selective cassette to create a linear fragment containing large homologous sequences to the upstream and downstream regions of the target lncRNAs. Following transformation and homologous recombination, the target lncRNA is replaced by the hph cassette.

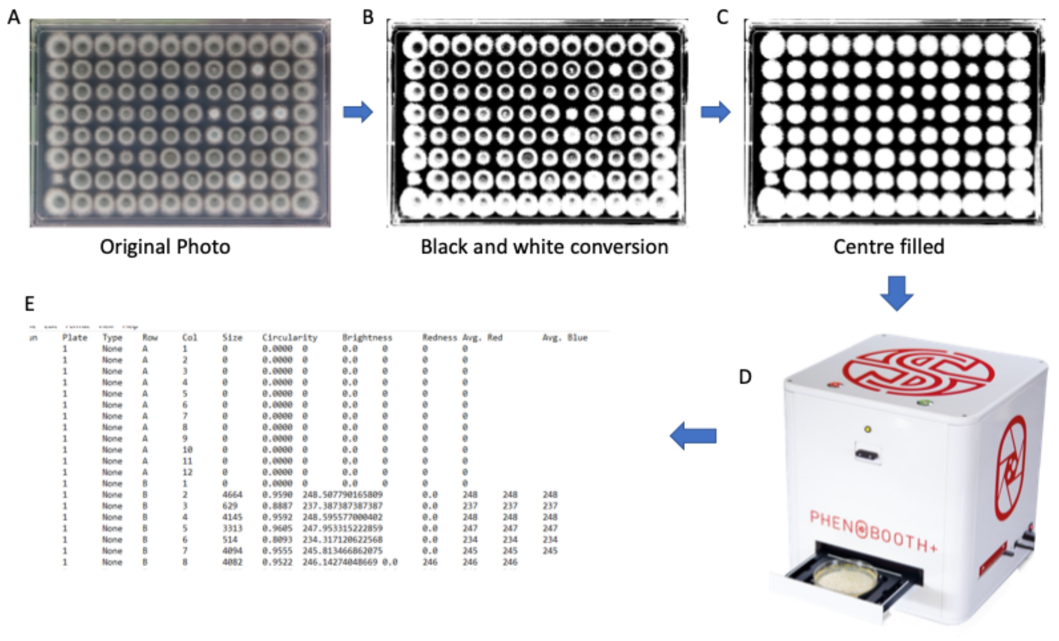

Supplementary Figure 4. Schematic of experimental method used for data analysis of colony sizes. A picture of each plate was taken in the same position with consistent light source. The original photo (A) was adjusted to black and white using photo processing software (Corel PaintShop Pro 2018) to optimize the analysis (B). The unevenly colored center of each colony was then uniformly filled in white (C) to improve the accuracy of the calculation of the colony size by the Phenobooth (Singer Instruments Ltd, UK) (D). The resulting colony size outputs (E) were used to calculate the relative fitness of each ncRNA KO.

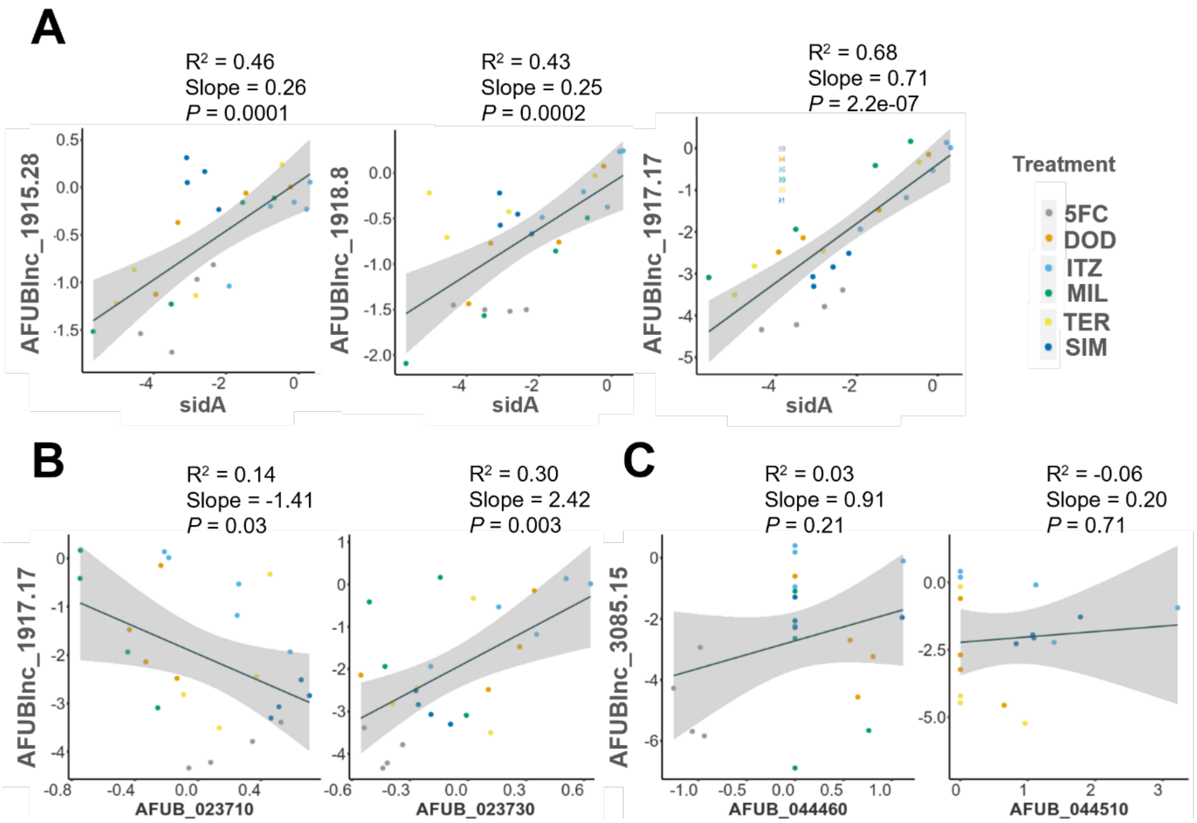

Supplementary Figure 5. There is significant co-expression observed between siderophore genes and proximal lncRNAs and similar correlation is not found between the lncRNAs and other nearby genes. A. Three lncRNAs (*AFUBlnc_1915.28*, *AFUBlnc_1918.8* and *AFUBlnc_1917.17*) are proximal to sidA and displayed significant co-expression with this gene ($p < 0.001$). Of these proximal lncRNAs, *AFUBlnc_1917.17* displays the highest correlated expression (log fold change) with sidA ($R^2 > 0.5$). **B.** *AFUBlnc_1917.17* displays considerably lower correlation ($R^2 < 0.5$) with the next two closest genes, *AFUB_023710* and *AFUB_023730*. In addition, the log fold change values of these genes are low (below 1). **C.** *AFUBlnc_3085.15* displays no significant correlation with the next two closest genes outside of the sidG cluster, *AFUB_044460* and *AFUB_044510*. Linear regression trend lines shown in black with grey bands indicating 95% confidence interval.

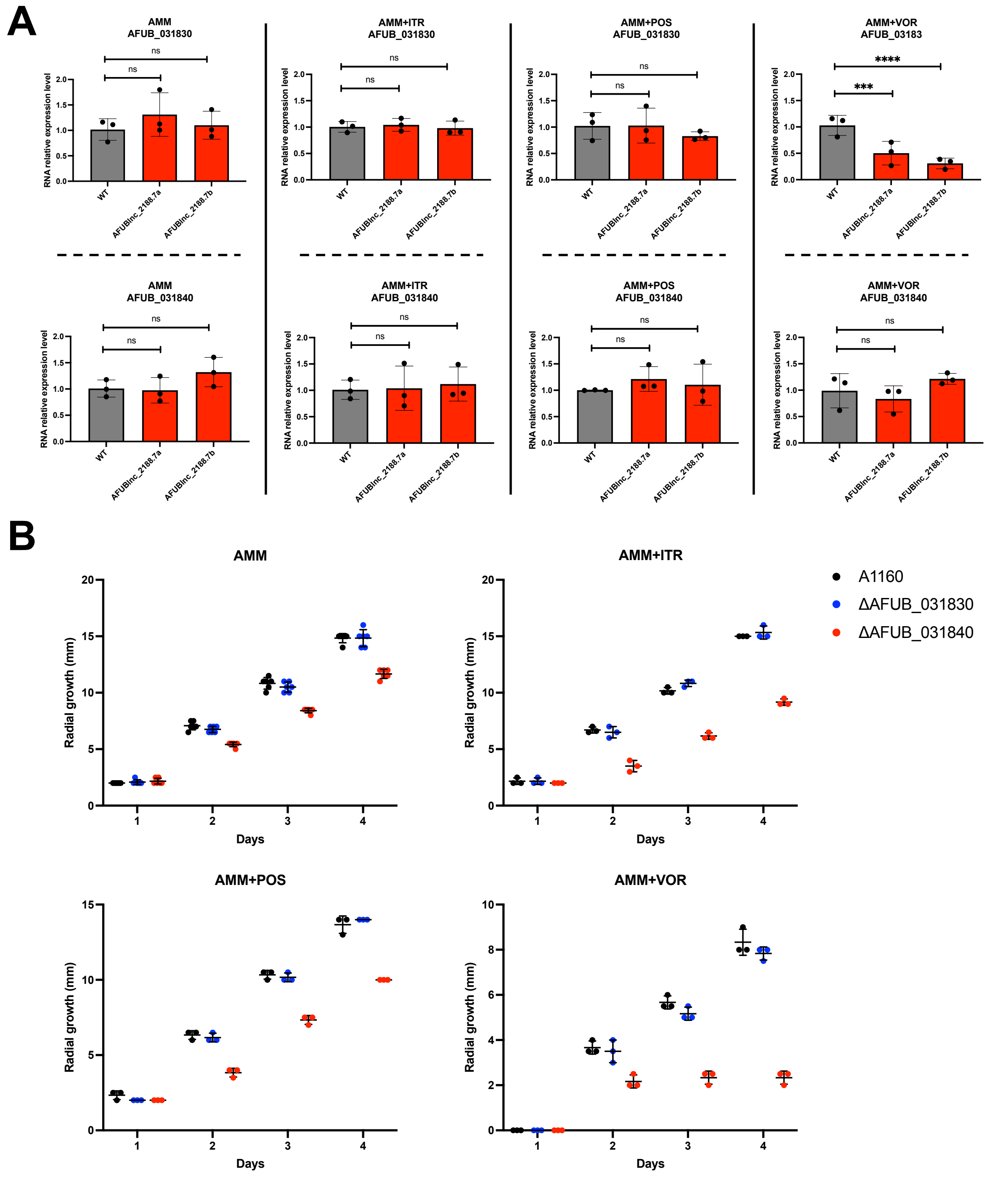

Supplementary Figure 6. The azole resistance phenotype in *ΔAFUBlnc_2188.7* is independent from its neighbouring genes. A. RNA expression levels of lncRNA *AFUBlnc_2188.7* flanking genes, *AFUB_031830* and *AFUB_031840*, was measured via qRT-PCR in both WT strain A1160 (grey) and lncRNA KO strains *ΔAFUBlnc_2188.7* (red) under standard media AMM and upon exposure to three azoles used in the screening (itraconazole, posaconazole, and voriconazole). The

relative RNA quantities of *AFUB_031830* and *AFUB_031840* were calculated by the ΔΔCt method using *actA* as reference. The expression of *AFUB_031830* or *AFUB_031840* in the lncRNA KO was compared to the expression in the WT. Each strain under each condition was analysed with three biological replicates (n=3), which are represented as individual dots on the bar chart. Error bars represent standard deviation. No significant differences in expression were observed for the downstream gene *AFUB_031840* between WT and lncRNA KO strains under any condition tested. Similarly, the upstream gene *AFUB_031830* showed no change in expression in the knockout strain under normal conditions or in the presence of itraconazole or posaconazole. A modest reduction (around -1 $\log_2$ fold change) in *AFUB_031830* expression was detected in the lncRNA KO strain in the presence of voriconazole. **B.** Radial growth phenotypes of *A. fumigatus* strains A1160 (parental isolate), *ΔAFUB_031830* and *ΔAFUB_031840* (strains deleted in *AFUBlnc_2188.7* neighbouring genes) in AMM and AMM supplemented with voriconazole (VOR, 0.25 mg/L), itraconazole (ITR, 0.0625 mg/L) or posaconazole (POS, 0.025 mg/L). Plates were incubated at 37◦C and colony radial growth measurements (mm) were taken for all strains at days 1, 2 3, and 4. Standard deviations represent averages of results from three technical repetitions. Dots represent individual values with lines representing averages and standard deviations of results from three (AMM+VOR, AMM+ITR, AMM+POS) or six (AMM) technical repetitions. ITR: itraconazole; VOR: voriconazole; POS: posaconazole. Source data for both panel are provided as a Source Data file.

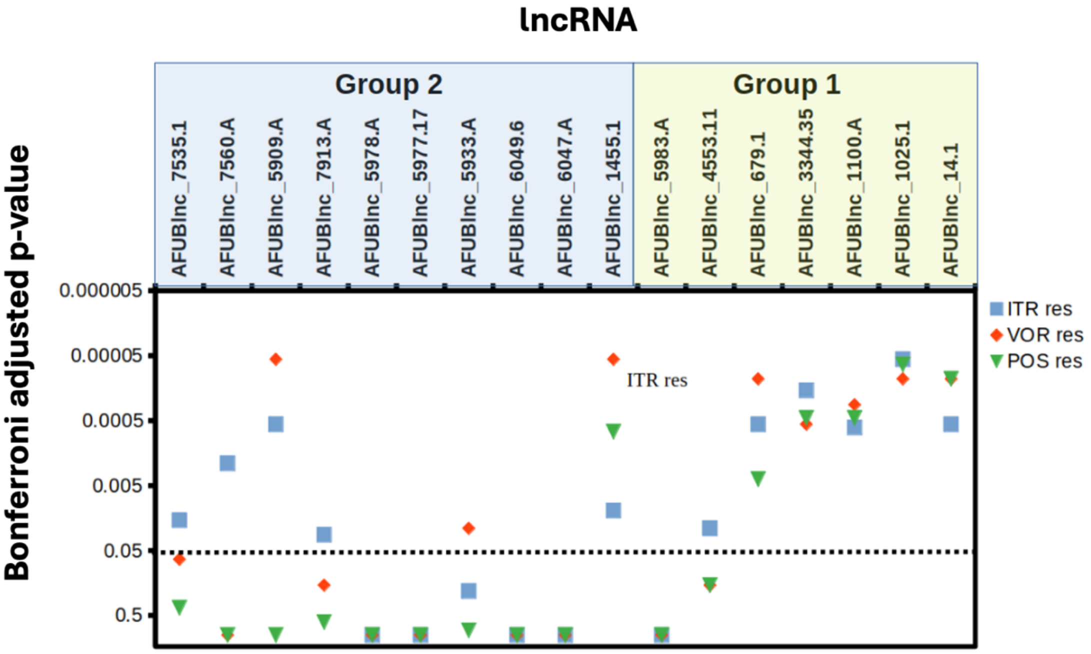

Supplementary Figure 7. Statistical association or presence or absence of lncRNA genes with resistance to azoles. Statistical association for each lncRNA gene was calculated using Fishers exact test. Of the 1187 lncRNAs 1030 were either completely present or completely absent in the test dataset which consisted of 130 publicly available *Aspergillus fumigatu*s genomes with complete azole MIC data. P-values were calculated and adjusted for multiple sampling using Bonferroni. The cut off for significance (Padj<0.05) is indicated by a dotted line. We note that the p-values do not completely match the groupings from the hierarchical clustering in Figure 4 with group 1 (*AFUBlnc_7535.1, AFUBlnc_7560.A, AFUBlnc_5909.A, AFUBlnc_7913.A, AFUBlnc_5978.A, AFUBlnc_5977.17, AFUBlnc_5933.A, AFUBlnc_6049.6, AFUBlnc_6047.A*) showing some association to ITR resistance and group 2 (*AFUBlnc_1455.1, AFUBlnc_5983.A, AFUBlnc_4553.11, AFUBlnc_679.1, AFUBlnc_3344.35, AFUBlnc_1100.A, AFUBlnc_1025.1, AFUBlnc_14.1*) mostly showing strong association to pan azole resistance.

Supplementary note

LncRNAs display coordinated expression with neighbouring protein coding genes

The ability of lncRNAs to play a role in azole response led us to identify further candidates that may be involved in azole susceptibility or resistance. To do this, we mapped data from an RNA-seq experiment which exposed strain A1163 to itraconazole at five MIC's (0.25, 0.5, 1, 2 and 4) to our newly generated transcriptome. Differential expression (DE) analysis was then performed in comparison to a no drug control. A total of 2,746 PCGs and 245 lncRNAs (intergenic=130, antisense=115), were found to have a significant response to itraconazole in at least one concentration (Fig. 2G). Clustering was performed to identify lncRNAs that have similar or opposing expression patterns with PCG's across dosage. Using a hierarchical k-means clustering approach we were able to generate 15 distinct groups that had parallel expression patterns (Fig. 2-I).

Using our clustering results, we were able to elucidate groups of PCGs and lncRNAs that are found to be localised within 5 kb of the genome and that have either a coordinated or divergent pattern of expression. We found 1,485 lncRNA-PCG pairs of neighbouring loci, with the majority of lncRNAs found to be upstream (n=1,043) of a PCG (Supplementary Data 10). Of these neighbours, 46 lncRNA-PCG pairs were found to be within the same cluster, indicating coordinated expression, whereas 16 were found to be inversely clustered, indicating divergent expression. Enrichment analysis found that there were no significantly over-represented ontologies in lncRNA-PCG pairs that share either similar or opposing expression profiles. Within the 46 pairs that exhibited a similar expression profile as their neighbouring lncRNA. We performed the same analysis on differentially expressed antisense lncRNAs and their "sense" PCGs (Supplementary Data 11). Within this study we identified a total of 492 sense/antisense (S/AS) pairs, of these we found 48 lncRNAs that are antisense to two PCGs and 3 that are antisense to three PCGs. A total of 14 S/AS pairs were clustered together and 6 were inversely clustered. Once again, neither of these pair sets were functionally enriched.

To understand whether these coordinated responses are due to the same regulatory elements controlling these loci we overlayed the binding sites of transcription factors from several studies. We found a total of 545 lncRNAs that have a transcription factor binding site within 200 bp upstream from their locus, from this we found 51 that do not have an adjacent PCG and 11 of these were found to be DE in response to itraconazole.

Cluster analysis found that three genes - *hapB*, *sidA* and *sidG* - had similar or opposing expression patterns to their corresponding lncRNA (see Supplementary Data 7, 10 and 11). The CCAAT-binding complex (CBC) subunit, HapB, has an upstream bidirectional lncRNA (*AFUBlnc_2633.12*) found to have an opposing expression pattern in response to itraconazole. The expression pattern of this lncRNA and its co-expression with hapB is distinct to the response to itraconazole, and not observed for other antifungal drugs (Supplementary Data 5).

The L-orthinine N5-oxygenase, SidA*,* is responsible for the first step in siderophore biosynthesis. The sidA gene is surrounded by lncRNAs, with one downstream (*AFUBlnc_1915.28*) and two upstream lncRNAs (*AFUBlnc_1917.17* and *AFUBlnc_1918.8*) (Fig. 3A). Although only the upstream bidirectional *AFUBlnc_1917.17* was classified in the same cluster as sidA (Supplementary Data 10, cluster 7), all three lncRNAs near *sidA* displayed co-down expression dose response patterns upon itraconazole treatment (Fig. 3B). The lncRNA *AFUBlnc_1917.17* was found to have a similar down expression pattern in response to all drugs excluding hygromycin, with all other drug responses identified in cluster 5 or 12 (Fig. 2J). Similar to the observation with itraconazole, *AFUBlnc_1917.17* and other neighbouring lncRNAs also displayed similar expression patterns to sidA in these drug datasets. There was significant correlation between sidA and *AFUBlnc_1917.17* log fold change values from all drug datasets excluding hygromycin (R^2 = 0.7, p = 2.2e-07) (Fig. 3C and Supplementary Figure 5A). There was also significant correlation between the other nearby lncRNAs and sidA LFC values, albeit with less apparent co-expression patterns (R^2 = 0.46, p = 0.0001 and R^2 = 0.43, p = 0.0002 for *AFUBlnc_1915.28* and *AFUBlnc_1918.8*, respectively) (Supplementary Figure 5A). No significant correlation was found between *AFUBlnc_1917.17* and the next two flanking genes (*AFUB_023710* and *AFUB_023730*) (Supplementary Figure 5B).

The fusarinine C acetyltransferase gene, sidG is located within one of the siderophore gene clusters which also includes mirB, estB and an ABC transporter (*AFUB_044470*)[57].The lncRNA *AFUBlnc_3085.15* is also within this gene cluster and is antisense to both sidG and estB (Fig. 3D). As well as sidG, earlier cluster analysis identified that *AFUBlnc_3085.15* also displayed similar itraconazole response pattern to mirB and the ABC transporter (all found in cluster 7) (Fig. 3E). Notably, *AFUBlnc_3085.15* does not display correlated expression with nearby genes which are outside of the SidG cluster (*AFUB_044460* and *AFUB_044510*) (Supplementary Figure 5C). When assessing the expression pattern of this lncRNA in other datasets, it was found to be down expressed in response to all drugs excluding hygromycin, with all other drug responses identified in either cluster 5 or 12 (Fig. 2J). As found with itraconazole, the lncRNA displayed a strikingly similar co-expression pattern to all the genes in the sidG cluster in response to simvastatin (Fig. 3E). Combining the data from all drug treatments excluding hygromycin demonstrated strong correlation between the LFC values of the lncRNA and each gene (Fig. 3F). Notably, the strongest correlation in LFC values was observed for the lncRNA with mirB and sidG ($R^2 = 0.96$, $p = 3.3e-18$ and $R^2 = 0.93$, $p = 5.1e-15$, respectively). Therefore, the novel lncRNA antisense to sidG and estB displays significant co-expression with all the genes in this siderophore-associated gene cluster.

The target of the azole antifungals, Cyp51A, has two lncRNAs found within its promoter region which are hence named Cyp51A-promoter lncRNA (CPL) for the lncRNA transcribed in sense to cyp51A (*AFUBlnc_4669.11*) and anti-CPL for the lncRNA described in the opposite direction (*AFUBlnc_4670.4*) (Fig. 3G). CPL covers all known regulatory binding motifs in the promoter, including the CCAAT-binding complex (CBC), HapX, SrbA[19], AtrR[58] and NctA/B[27]. Anti-CPL is shorter than CPL and contained entirely within it, and notably excludes the significant regulatory section near the TR34 region. In untreated culture, CPL and anti-CPL are expressed at above median lncRNA levels (3.3 TPM), 28.6 and 11.7 TPM, respectively. An example of the transcriptional profile of Cyp51A and its upstream lncRNAs is shown in Fig.3H. Transcription from the lncRNA locus upstream of Cyp51A was validated using qRT-PCR (Fig. 3J). In response to itraconazole, CPL is significantly differentially expressed with a LFC of 1.7 at 4-fold MIC. Clustering of gene and lncRNA responses upon

itraconazole treatment did not identify correlated expression between either lncRNA and Cyp51A. When assessing the behaviour of the lncRNAs and Cyp51A in the other drug datasets, it is apparent that CPL was consistently upregulated in response to drug treatment and showed distinct dose response expression patterns to Cyp51A (Fig. 3I).
