# Supplementary material for: Genome-wide discovery and phenotyping of non-coding transcripts in A. fumigatus reveals lncRNAs with a role in antifungal drug sensitivity
Source: Nat Commun. 2026 Feb 11;17:1832. doi: 10.1038/s41467-026-68543-9 (PMC12921299; doi:10.1038/s41467-026-68543-9)
Supplement: Supplementary file 2 — Description Of Additional Supplementary File [file 41467_2026_68543_MOESM2_ESM.pdf]

Description of Additional supplementary files

Title: Supplementary data 1 - lncRNA annotation.xlsx.
Description: Annotation for the maually curated set of 1089 novel lncRNAs. Annotation coordinates refer to lncRNA mapped against *A. fumigatus* A1163 genome sequence obtained from Ensembl Fungi (Aspergillus_fumigatusa1163. ASM15014v1.dna.nonchromosomal.fa). The type of lncRNA (antisense or intergenic) is indicated. For antisense lncRNA, its gene partner is also noted.

Title: Supplementary data 2 - BLAST output.xlsx
Description: BLASTn output of aligned lncRNA against 127 *Aspergillus* genomes.

Title: Supplementary data 3 - Itraconazole DE.xlsx
Description: Differential expression and hierarchical k-means clustering of expressed RNA from *A. fumigatus* after exposure to itraconazole at five different dosages.

Title: Supplementary data 4 - Multi-drug DE.xlsx
Description: Differential expression and hierarchical k-means clustering of expressed RNA from *A. fumigatus* after exposure to multiple drugs at four different dosages.

Title: Supplementary data 5 - Multi-drug clust.xlsx
Description: Clustering of transcripts with similar expression profiles across multiple drugs.

Title: Supplementary data 6 - Gene search list.xlsx
Description: Azole-associated genes used for co-localised lncRNA search.

Title: Supplementary data 7 - co-local. lncRNA.xlsx
Description: lncRNA co-localised with azole associated genes.

Title: Supplementary data 8 - Phenotypic data.xlsx
Description: Summary of validation for the phenotypic screening.

Title: Supplementary data 9 - Primer sequences.xlsx
Description: Primers used for the deletion of lncRNAs.

Title: Supplementary data 10 - Neighbour data.xlsx
Description: Neighbour analysis of protein-coding genes and lncRNA with their distances and clustering profiles.

Title: Supplementary data 11 - Sense-antisense.xlsx
Description: Summary of protein-coding genes and lncRNA that exhibit sense-antisense pairings with their intersections and clustering profiles.

Title: Supplementary data 12 - List of genomes with known azole MICs.xlsx
Description: List of genomes with known azole MICs. Genomes obtained from NCBI SRA archive with complete azole MIC data.
